# Evaluation on the Pharmacological Effect of Traditional Chinese Medicine SiJunZiTang on Stress-Induced Peptic Ulcers

**DOI:** 10.1155/2013/186076

**Published:** 2013-06-12

**Authors:** Chiu-Mei Chen, Chien-Ying Lee, Po-Jung Lin, Chin-Lang Hsieh, Hung-Che Shih

**Affiliations:** ^1^Department of Neurology, Chung Shan Medical University Hospital, Taichung, Taiwan; ^2^Institute of Medicine, Chung Shan Medical University, Taichung, Taiwan; ^3^School of Dentistry, College of Oral Medicine, Chung Shan Medical University, Taichung, Taiwan; ^4^Department of Pharmacology, Chung Shan Medical University, No. #110, Section 1, Chien-Kuo North Road, Taichung 40201, Taiwan; ^5^Department of Pharmacy, Chung Shan Medical University Hospital, Taichung, Taiwan; ^6^Department of Pharmacology, Tokyo Medical University, Tokyo, Japan

## Abstract

*Purpose*. To explore the effects of SiJunZiTang (SJZT) on central neurotransmitters and the inhibition of HCl hypersecretion, along with the role of the vagus nerve. From this, the effects of SJZT and its constituent ingredients on inhibiting stress-induced peptic ulcers will be determined. *Methods*. Methods used to determine SJZT's effectiveness included (1) measuring the antipeptic ulcer effects of varying combinations of the constituents of SJZT; (2) evaluations of monoamine (MA) level in the brain; and (3) measuring the effects of longer-term SJZT treatment. *Results*. Comparing the control and experimental groups where the rats' vagus nerves were not cut after taking SJZT orally (500 mg/kg and 1000 mg/kg), the volume of enterogastric juice, free HCl and total acidity all reduce dose-dependently. The group administered SJZT at 1000 mg/kg showed significant reductions (*P* < 0.05). For the experimental groups where the vagus nerves were cut, a comparison with the control group suggests that the group receiving SJZT (500 mg/kg) orally for 21 days demonstrated a cure rate of 34.53%. *Conclusion*. The results display a correlation between the therapeutic effects of SJZT on stress-induced peptic ulcers and central neurotransmitter levels. Further to this, SJZT can inhibit the hypersecretion of HCl in the stomach, thus inhibiting stress-induced peptic ulcers.

## 1. Introduction

The occurrence of self-reported ulcer over a nine-year period was more likely in subjects who reported any of several concrete life stressors or psychological distress at baseline. The ulcerogenic effects of stress have been shown to be robust enough to survive adjustment for behavioral and physical confounding factors [1]. True stress ulcers are primarily superficial gastric fundus lesions that occur in the clinical setting of severe shock, trauma, burns, and sepsis, especially peritonitis. 

SiJunZiTang (SJZT) is a basic prescription, consisting of four components:* Ginseng Radix, Poria Cocos, Atractylodis Rhizoma*, and *Glycyrrhizae Radix*. SJZT is a common Chinese herbal prescription, tonifying the spleen and stomach, is traditionally used for the treatment of gastrointestinal diseases in oriental countries. 

The antiulcer activities of SJZT also have been mentioned in recent research [2]. However, the antiulcer mechanism of SJZT is not clear. Several studies indicated the CNS in modulating gastrointestinal function and response to injury [3, 4]. Dopamine (DA) plays a critical role in the protection of gastric mucosa and is mediated through corresponding receptors [5]. Dopamine D_1_ (central)/DA_1_ (peripheral) receptors are believed to influence gastrointestinal functions and pathologies. Centrally administered D_1_ agonists exert effects of greater magnitude than when administered peripherally [3, 4]. The hypothesis of a “brain-gut axis” was proposed to account for the growing body of evidence showing that a variety of peptides, neurotransmitters and neuromodulators profoundly affect not only gastric secretory responses but also gastric and duodenal ulcerogenesis [3, 4].

The goal of this study was to better understand the correlation between the inhibitory effects of SJZT on hypersecretion of free HCl, and how the pharmacological effect of SJZT could inhibit stress-induced peptic ulcers, and whether this was associated with the central neurotransmitters, such as dopamine (DA), norepinephrine (NE), and serotonin (5-hydroxytryptamine, 5-HT), and the different combinations of its ingredients on stress-induced peptic ulcers.

## 2. Materials and Methods

### 2.1. Materials

#### 2.1.1. Experimental Animals

The study used healthy male Wistar Kyoto (WKY) rats with a body weight of 180 ± 20 g, obtained from the National Laboratory Animal Breeding and Research Center for experiments. The rats were fed with maintenance diet (Altromin 1320) and housed in a room of specific pathogen-free (SPF) facility of the Experimental Animal Center of Chung Shan Medical University, in which the room temperature maintained at 25 ± 1°C, relative humidity 55 ± 5%, air change rate (ventilation rate) 12 times/hour, and the artificial light application time was from 07:00 to 19:00. The rats were kept in the room for at least 7 days before the experiment to allow them to adapt to the environment. 

Our study protocol and experimental design were approved by the Institutional Animal Care and Use Committee (IACUC), Chung Shan Medical University Experimental Animal Center.

#### 2.1.2. Medication Used

SiJunZiTang (SJZT) herb formulas were prepared according to the official compendia “Tai Ping Hui Min Her Ji Jyu Fang” of the Song Dynasty in China. These included *Ginseng Radix*, *Poria Cocos*, *Atractylodis Rhizoma,* and *Glycyrrhizae Radix*. The mixing ratio of the ingredients was 1 : 1 : 1 : 1.

**Figure 1:**
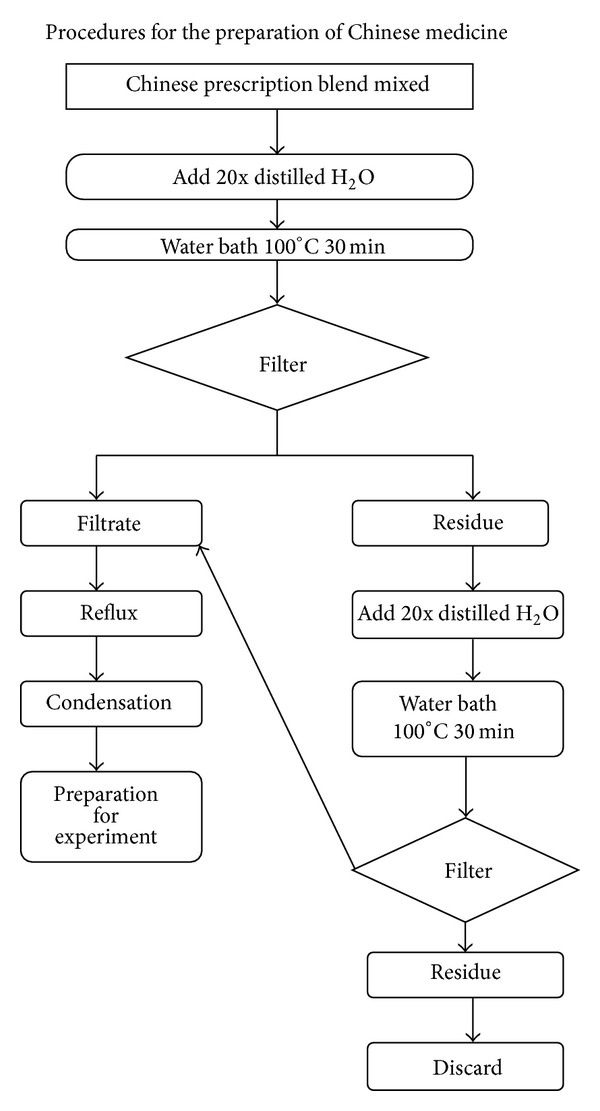
Procedures for the preparation of SJZT.

**Figure 2:**
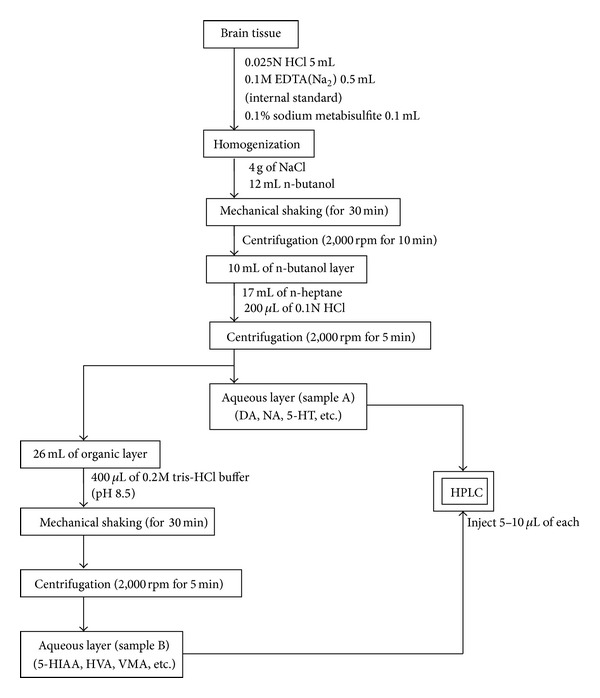
Flow chart of the preparation process for monoamine.

All herbal ingredients were of a high quality, without damage by moths or worms, preserved and frozen in alcohol-free fluid extracts. The preparation procedure is illustrated in Figure 1. SJZT extract was procured from a good manufacturing practices (GMP) supplier. Purity and contaminant tests for the presence of toxic metals, pesticide residues, mycotoxins, and microorganisms were performed in our study. Qualitative chemical fingerprint analysis and quantitation of marker compounds of these traditional Chinese medicines (TCM) were carried out with high-performance liquid chromatography (HPLC). Extraction and manufacture of these TCM were carried out under GMP and in accordance with The Japanese Pharmacopoeia 15th edition (JP15). Chemical standardization was performed with liquid chromatography-mass spectrometry (LC-MS) analysis. Stability of the formula was monitored with HPLC in real time. A HPLC flow chart is illustrated in Figure 2.

The SJZT was maintained in a refrigerator under 4°C and was prepared as a medicament (10 mL/kg) with dist. H_2_O. The medicament would be used for oral administration (p.o.) or for administration through duodenum (i.d.). 

DA, NA, 5-HT, and the metabolite VMA (vanillylmandelic acid), HVA (homovanillic acid), and 5-HIAA (5-hydroxyindole acetic acid) were adopted for analysis on the content of monoamine in brain. All the reagents were purchased from Sigma.

The HPLC-grade distilled water used in this experiment was purchased from a pharmaceutical factory.

#### 2.1.3. Apparatuses Used

A barrel which was made of a 4 cm the diameter wire net was used for stressed peptic ulcers induced by water immersion. With regard to the analysis of monoamine content changed in brain, a Model 440 HPLC Absorbance Detector (uv 280 nm, Model 717Autosample) with Data Model 746 produced by Waters was used. 

A Stereomicroscope (Leica WILD M3Z) was used for detecting the ulcerative areas.

### 2.2. Methods

#### 2.2.1. Blockade of Gastric Vagus Nerves and Relevant Effects

In this experiment, we fixed a rat on the operation panel and anesthetized with ether (WAKO of Japan), and then a cut was made from the point about 1.5 cm below the xiphoid and separated along the chest midline to the lower abdomen. The stomach of the rat was pulled out with apex circle-shaped tweezers. Next step, we separated the vagus nerves that were close to the cardia and nearby both sides of the esophagus, dissociated the nerves with a thin hook-shaped glass bar, and used the ophthalmological surgical fine scissors to cut off the vagus nerves. Finally, the stomach was put back into the rat's body and then sutured. These rats would be used for experiments until recovery. A total of 10 rats were used during the experiment.

#### 2.2.2. Determination on Secretion of Gastric Juice and Relevant Effects

8 healthy rats and 8 gastric vagus nerves cut-off rats were used. In the experiment, the rats were fed with dist. H_2_O (5.0 mL/kg) and SJZT (500 mg/kg and 1000 mg/kg) by oral administration, respectively. 30 minutes after feeding, the rats were anesthetized with ether. Then we cut the belly open for exposing the pylorus, and a 100% cotton silk was used to ligate the junction of the pylorus and the duodenum. Finally the stomach was put back into the abdomen and we sutured the wound. 8 hours later, the animals were sacrificed and we cut the bellies open again for removing their stomachs out. Before taking the stomach out, both the cardia and the pyloric sphincter were nipped with hemostatic forceps. Finally, a cylinder was used to measure the volume of gastric juice.

#### 2.2.3. Determination on Free HCl and Total Acidity in Gastric Juice and Relevant Effects

After processing the procedures stated in Section 2.2.2, we collected the gastric fluid, and then the fluid was centrifuged at a speed of 2000 rpm for 10 minutes. A 10 mL supernatant was taken out, placed into a beaker, and added with 2 drops of indicator A and B, respectively (A: 0.5% dimethylaminoazobenzene alcohol solution, B: 1% phenolphthalein alcohol solution). Then 0.1 N NaOH was used for titration. The amount of titrant used was recorded as A mL when the solution presented orange color. The amount of titrant consumed was recorded as B mL when the orange color solution turned into roseate color. According to A and B, the free HCl and the total acidity in the gastric juice were calculated. The unit of acidity is mEq/mL.

#### 2.2.4. Antipeptic Ulcer Effects of Different Combinations of Ingredients of SJZT

The different combinations of ingredients of SJZT, *Radix Ginseng* + *Glycyrrhizae Radix, Atractylodis Rhizoma* + *Glycyrrhizae Radix, Radix Ginseng* + *Poria Cocos, Atractylodis Rhizoma* + *Poria Cocos, Radix Ginseng* + *Atractylodis Rhizoma, Poria Cocos* + *Glycyrrhizae Radix, Poria Cocos* + *Atractylodis Rhizoma* + *Glycyrrhizae Radix, Radix Ginseng* + *Atractylodis Rhizoma* + *Radix Glycyrrhizae, Poria Cocos* + *Radix Ginseng* + *Glycyrrhizae Radix, Poria Cocos* + *Atractylodis Rhizoma* + *Radix Ginseng, and Radix Ginseng* + *Poria Cocos* + *Atractylodis Rhizoma* + *Glycyrrhizae Radix* + dist. H_2_O, were made into medicines 500 mg/kg (B.W.), respectively. All possible combinations of the constituent ingredients of SJZT were investigated. The medicines were prepared into doses 5.0 mL/kg (B.W.) and fed to the experimental rats by oral administration. 30 minutes later, the rats were placed in a water immersion restraint device designed by Takagi and Okabe [6] and immersed up to their xiphoid for 8-hour water bath at the temperature of 20 ± 1°C. Finally the animals were sacrificed and their stomachs were taken out. After sacrificing the animals, their stomachs were removed out, washed with 37°C normal saline, inflated with 37°C 10 mL 2% formalin, and fixed in the formalin solution for 5 minutes. The stomachs were cut, opening from the pylorus to the cardia along the greater curvature and washed in normal saline. After blotting up with filter papers, we observed the stomachs with a stereo microscope (Leica WILD M32) to determine the ulcerative areas, and then we measured and compared the total gastric ulcer length.

#### 2.2.5. Effects of SJZT on Long-Term Continuous Treatment for Peptic Ulcers

We placed 40 healthy male Wistar rats in a water immersion restraint device designed by Takagi for 7 days to induce stress gastric ulcers. Then 20 rats were sacrificed randomly to check the gastric ulcerative areas. After checking and verifying all the 20 rats' ulcerative areas, we could identify the others as morbid rats with stress-induced gastric ulcer. Then 20 rats were randomly divided into Groups A and B with 10 rats in each group. The rats in Group A took SJZT (500 mg/kg/day) orally at 08:00 A.M. for 14 days. The rats in Group B took SJZT (500 mg/kg/day) orally at 08:00  A.M. for 21 days. Then the rats were sacrificed and their stomachs were taken out when the terms expired. Afterwards the stomachs were cleaned up with normal saline and were inflated with 10 mL formalin (2%) at 37°C. All the procedures were mentioned in Section 2.2.4.

#### 2.2.6. Effects of SJZT on Monoamine in Brains of the Rats

A total of 6 rats from each group were used in the experiment. The rats heads were beheaded in 1 hour after feeding with SJZT (500 mg/kg) and 5.0 mL/kg dist. H_2_O by oral administration. Then we removed their brains and quickly separated the cortex and brain stem (including the lower part of brain stem) in a cold room at 4°C. According to the approach of Shibuya et al. [7], HPLC of Waters Associates was used to determine the content of DA, NE, and 5-HT as well as relevant typical metabolite involving homovanillicacid (HVA), 3-methoxy-4-hydroxy-phenyl-ethyleneglycol (MHPG), and 5-hydroxy-indole acetic acid (5-HIAA) in each part. Finally the data collected were compared with the control group. Considering that the content of MA in brain might be affected by time, the animals were sacrificed at 11:00  A.M. in the experiment.

#### 2.2.7. Statistical Method of Data

Data obtained in the experiments were expressed in the format of mean ± S.E., and the statistical method of one-way ANOVA was adopted for comparisons between experimental groups and control groups. *P* < 0.05, *P* < 0.01, and *P* < 0.001 refer to the significant difference statistically.

## 3. Results

### 3.1. Determination on Secretion of Gastric Juice and Relevant Effects

As Table 1 shows, in the group vagus nerve not yet cut off, the comparison between the control group fed with dist. H_2_O (5.0 mL/kg) orally and the experimental groups fed with SJZT (500 mL/kg and 1000 mg/kg individually) orally suggests that gastric juice reduces dose dependently. The group fed with SJZT (1000 mg/kg) presents an effective significant difference (*P* < 0.05). For the nerve transection groups, both the control group and the experimental group, the amount of gastric juice is less than the groups where the nerves were not cut reducing down to approximately 1/3.

### 3.2. Determination on Free HCl and Total Acidity in Gastric Juice and Relevant Effects

As Table 1 shows, The vagus nerve was not cut group; the comparison between the control group fed with Dist. H_2_O (5.0 mL/kg) orally and the experimental groups fed with SJZT (500 mL/kg and 1000 mg/kg individually) orally suggests that free HCl and total acidity in gastric juice reduce dose dependently. The experimental group fed with SJZT (1000 mg/kg) presents an effective significant difference (*P* < 0.05). For the nerve transection groups, both the control group and the experimental group, the gastric juice within free HCl and total acidity, are less than the groups where the vagus nerves were not cut, only the group administrated with SJZT (1000 mg/kg) presents a significant reduction (*P* < 0.05) in free HCl.

### 3.3. Antipeptic Ulcer Effects of Different Combinations of Ingredients of SJZT

**Figure 3:**
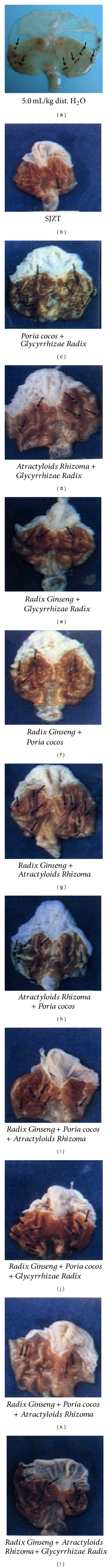
Effects of SJZT and relevant ingredients on stress-induced peptic ulcers.

As Table 2 and Figure 3 show, total peptic ulcer length of the control group that took dist. H_2_O (5.0 mL/kg) orally is 40.57 ± 0.78 mm; total peptic ulcer length of the experimental group that took SJZT (500 mg/kg) orally is 14.10 ± 0.51 mm (*P* < 0.001). For the group of *Radix Ginseng *+ *Glycyrrhizae Radix* (500 mg/kg), total peptic ulcer length is 15.32 ± 0.64 mm (*P* < 0.01). For the group of *Atractylodis Rhizoma* + *Glycyrrhizae Radix *(500 mg/kg), total peptic ulcer length is 20.29 ± 0.69 mm (*P* < 0.05). For *Radix Ginseng* + *Poria Cocos *(500 mg/kg) group, total peptic ulcer length is 21.50 ± 0.55 mm (*P* < 0.05), and for *Poria Cocos* + *Atractylodis Rhizoma* + *Radix Ginseng *(500 mg/kg) group, total peptic ulcer length is 15.94 ± 0.29 mm (*P* < 0.05).

### 3.4. Effects of SJZT on Long-Term Continuous Treatment for Peptic Ulcers

As Table 3 shows, for the experimental group that was administrated SJZT (500 mg/kg) for 14 days successively, total peptic ulcer length is 97.5 ± 1.88 mm. For the experimental group that was administrated SJZT (500 mg/kg) for 21 days successively, total peptic ulcer length is 78.67 ± 1.47 mm (*P* < 0.05). Comparing with the control group that was administrated Dist. H_2_O (5.0 mL/kg), prolong administration SJZT (500 mg/kg) can be obtained 34.53% benefits for the treatment of stress-induced peptic ulcers.

### 3.5. Effects of SJZT on Monoamine in Brain of Rat

As Tables 4(a) and 4(b) show, whether to give SJZT 500 mg/kg or SJZT 1000 mg/kg, NA, DA, and 5-HT contents in the experimental group of rat brain cortex or brain stem are lower than those in the control group and present a dose-dependent reduction (*P* < 0.01). Monoamine metabolite including VMA, HVA, and 5-HIAA content of the sites as well as the difference in the amount administered has different levels of change. 

## 4. Discussion

Psychological stress is not only empirically associated with ulcers but is a very plausible risk factor for ulcer disease. Gastric acid output is correlated with psychological distress in patients with and without ulcers and increased enormously during intense military training [1]. Peptic ulcer disease is a deep gastrointestinal erosion disorder that involves the entire mucosal thickness and can even penetrate the muscular mucosa. 

Numerous natural products have been evaluated as therapeutics for the treatment of a variety of diseases. These sources of products usually are derived from plants and animals that contain active constituents such as alkaloids, flavonoids, terpenoids, and tannins. The alkaloids are natural nitrogen-containing secondary metabolite mostly derived from amino acids and found in about 20% of plants [8]. Intragastric or intradermal administration of an ethanol extract of *Radix Ginseng* to rats decreased histamine, pentagastrin, carbachol, and vagal stimulation-induced gastric secretion and inhibited gastric ulcers induced by stress or by pyloric ligation [9–11]. *Poria Cocos *is used as a diuretic, sedative, and tonic; Triterpene acids and polysaccharides are the principal ingredients of *Poria Cocos *that are responsible for diverse bioactivities, including antitumor, anti-inflammatory, antioxidant, and antiemetic effects [12]. The dried rhizome of *Atractylodes macrocephala *Koidz is used as a digestive and a tonic, in which volatile oils, polysaccharides, sesquiterpenes, and flavonoids were identified with anti-inflammatory, hypoglycemic, and gastrointestinal inhibitory effects [12]. One study showed that the acetylene compound from *Atractylodes rhizome* significantly suppressed xanthine oxidase (X.O.) activity in the stomach tissue. The suppressive effects of this compound on lesion formation induced by indometacin and ischemia-reperfusion injury models would thus appear partly due to the inhibition of X.O. activity in the stomach tissue [13]. The antiulcer activity of *Glycyrrhizae Radix *has been demonstrated both experimentally and clinically. Intraperitoneal, intraduodenal oral administration of aqueous or alcoholic extracts of *Glycyrrhizae Radix *reduced gastric secretions in rats, and it inhibited the formation of gastric ulcers induced by pyloric ligation, aspirin, and ibuprofen [14]. Glycyrrhizin and its aglycone (glycyrrhetic acid, enoxolone), two of the active constituents of *Glycyrrhizae Radix*, both have antiphlogistic activity and increase the rate of mucus secretion by the gastric mucosa. Deglycyrrhizinated liquorice (97% of glycyrrhizin is removed) effectively treated stress-induced ulcers in animal models [14–16]. The mechanism of antiulcer activity involves acceleration of mucin excretion through increasing the synthesis of glycoprotein at the gastric mucosa, prolonging the life of the epithelial cells and antipepsin activity [14].

Stress has been shown to alter normal dopaminergic neurotransmission, and exposure to stress profoundly increases the dopaminergic activity and induces relevant adaptive response of DA receptors in specific brain regions. Stress also activates the hypothalamus-pituitary-adrenal (HPA) axis and releases glucocorticoids. The stress-induced adaptation of brain DA function involves receptors, and it has also been demonstrated that DA receptor densities are affected by altered extracellular DA levels [17]. The great majority of studies in vivo have reported that DA or dopaminergic compounds inhibit the secretion of gastric acid or pepsin, stimulate the secretion of mucus or bicarbonate, and regulate mucosal blood flow [5]. Smooth-muscle cells express 5-HT_1_ and 5-HT_2_ effector serotonin receptors. Intramural ganglionar neurons and enterochromaffin cells have surface 5-HT_3_ and 5-HT_4_ receptors. Through these receptors, 5-HT regulates the contractile activity of smooth muscles. Serotonin induces contractions of the smooth-muscle cells of the fundal compartment of the stomach during reaction with 5-HT_2B_ receptors [18]. During ulcer relapse, we noted significantly raised levels of NA, DA and free 5-HT. After healing ulcer, significant reductions in NA, DA, free 5-HT, and significant increases in platelet serotonin values were observed. NA remained higher and platelet serotonin lower, both significantly more than the normal. The results demonstrate that some baseline autonomic system imbalance exists in ulcer patients, amplified and accentuated during relapse [19].

The secretion of gastric acid (HCl) is intimately related to peptic ulcer disease. Gastrin G-cells and somatostatin D-cells are important regulators of gastric acid secretion, and alterations in their relative numbers may play a key role in gastroduodenal disease [20]. In humans, gastrin is a peptide hormone that stimulates secretion of gastric acid (HCl) by the parietal cells of the stomach and aids in gastric motility. In this study, the vagus nerves of experimental animals were not cut; their secretion of gastric juice, free HCl, and total acidity in gastric juice present a dose-dependent reduction no matter they were fed with Dist. H_2_O or SJZT (500 mg/kg). With regard to the group that was fed with SJZT (1000 mg/kg), a significant inhibition effect (*P* < 0.05) on secretion of gastric juice, free HCl, and total acidity in gastric juice is presented; but other groups have not indicated any valid significant difference. As the experimental animals whose vagus nerves were cut, although their secretion of gastric juice, free HCl, and total acidity in gastric juice increased or decreased to different extents, there is no any meaningful significant difference. The only exception is that the group fed with SJZT (1000 mg/kg) presents a remarkable inhibition effect on free HCl (*P* < 0.05) and 1/3 secretion volume of gastric juice, compared with the groups which vagus nerves were not cut. Therefore, we confirm the same findings as demonstrated in the groups of which vagus nerves were not cut; SJZT has the effect of inhibiting free HCl in gastric juice. SJZT may inhibit G-cell to secret gastrin, and free HCl in gastric juice can be reduced.

We also found that the effect of SJZT on content change of neurotransmitters in nervus centralis SJZT could reduce the content of 5-HT and DA in brain cortex and brain stem. This is consistent with SJZT having a more pronounced sedatory effect than Diazepam as concluded in our previous research report [21]. This result is consistent with after healing ulcer; significant reductions of DA, 5-HT were observed [19]. The results demonstrated that SJZT has antiulcer effect.

Chinese traditional prescription was made according to monarch, minister, assistant, and guide mode. This mode connects the modern thoughts of treating a disease by differentiating the disease and the symptoms, also determining a formula according to both the disease and syndrome. The proportions used in this study are based upon the source book of SJZT [Tai Ping Hui Min Her Ji Jyu Fang]. No changes have been made to this traditional predetermined formulation with *Radix Ginseng, Poria Cocos*, *Atractylodis Rhizoma,* and *Glycyrrhizae Radix* combined in equal measures.

In traditional Chinese culture, *qì* (also *chi*) is an active principle forming part of any living thing. *Qi* is frequently translated as “life energy,” “life force,” or “energy flow.” Qi is the central underlying principle in traditional Chinese medicine. *Radix Ginseng* offers a sweet flavor and warming effects on the spleen and lung meridians, invigorating primordial *qi*, and is the principal herb in this formula. *Atractyloids Rhizoma works *as an assistant herb with a sweet and bitter flavor and offers warming properties and strengthens the spleen in order to dry dampness and invigorates the stomach to harmonize the middle. *Poria Cocos *with a sweet flavor, excretes damp and strengthens the spleen. It assists *Atractyloids Rhizoma* in invigorating the spleen function to remove dampness and is an adjuvant herb. *Glycyrrhizae Radix* with a sweet flavor and a warming property, replenishes *qi* and is used as a guiding herb to harmonize all herbs in the formula [22].

One of the purposes of the study is to determine the effects of the differing combinations of the constituent parts of SJZT. Every possible combination was tested through from the testing of individual ingredients working alone to all possible combinations of the three ingredients (in equal measure). In our study, we could observe that antiulcer effect was significant in *Radix Ginseng* + *Glycyrrhizae Radix *(*P* < 0.01), *Atractylodis Rhizoma* + *Glycyrrhizae Radix* (*P* < 0.05), *Radix Ginseng* + *Poria Cocos *(*P* < 0.05), *Poria Cocos* + *Atractylodis Rhizoma* + *Ginseng Radix *(*P* < 0.05), and in SJZT group (*P* < 0.001). This represents that the composing formula mode is an important meaning for promoting the research of treating diseases by differentiating integrated syndrome and symptom, enriching composing formula theory, and creating new formula in the clinic. As gastric mucosa contains mucous glycoprotein which is a kind of polymeric glycoprotein, it has the protection function to avoid autopepsia of pepsin which may cause peptic ulcer. Ginsenoside Rb1 was a component from *Radix Ginseng* which was investigated for its antiulcer effect [23]. Glycyrrhizin was a component from *Glycyrrhizae Radix* which was investigated for its antiulcer effect [24]. *Radix Ginseng* and *Glycyrrhizae Radix* of SJZT contain the ingredients such as ginsenoside and glycyrrhizin those are the main predecessors for synthesizing glycoprotein. The theory also is verified in the experiment that application of the combination of *Radix Ginseng* and *Glycyrrhizae Radix* achieved the curative effect on stress-induced peptic ulcers similarly to SJZT.

We conclude that SJZT not only reduces the content of free HCl in gastric juice to inhibit the attack factors and stimulate gastric mucosa secreting gastric mucus to protect the defense factors of gastric mucosa, but also reduces the content of 5-HT and DA in brain cortex and brain stem. SJZT has an excellent preventive and therapeutic effect on stress-induced peptic ulcers. SJZT could be considered as an alternative for the treatment of stress-induced peptic ulcers.

## Figures and Tables

**Table 1:** Effects of SJZT on volume of gastric juice, free HCl, and total acidity of gastric acid for rats of vagus nerves were cut and not cut.

Types	Groups	UN-vagotomy	Vagotomy
Volume	Dist. H_2_O 5.0 mL/kg	9.92 ± 0.62 mL	3.82 ± 0.30 mL
	SJZT 500 mg/kg	6.21 ± 0.93 mL	1.82 ± 0.52 mL
	SJZT 1000 mg/kg	5.43 ± 0.47* mL	2.89 ± 0.64 mL

Free HCl	Dist. H_2_O 5.0 mL/kg	49.67 ± 3.11 mEq/mL	1.73 ± 0.51 mEq/mL
	SJZT 500 mg/kg	25.06 ± 2.80 mEq/mL	1.65 ± 0.63 mEq/mL
	SJZT 1000 mg/kg	21.02 ± 2.02* mEq/mL	0.91 ± 0.26* mEq/mL

Total acidity	Dist. H_2_O 5.0 mL/kg	81.17 ± 4.08 mEq/mL	7.18 ± 0.87 mEq/mL
	SJZT 500 mg/kg	74.03 ± 2.55 mEq/mL	6.59 ± 0.96 mEq/mL
	SJZT 1000 mg/kg	39.31 ± 3.06* mEq/mL	7.82 ± 0.82 mEq/mL

*P < 0.05; *N* = 6; mean ± S.E.; peroral, control group: Dist. H_2_O 5.0 mL/kg (B.W.)

**Table 2:** Effects of SJZT and relevant ingredients on stress-induced peptic ulcers.

Number	Groups	Results
a	5.0 mL/kg Dist. H_2_O	40.57 ± 0.78 mm
b	SJZT	14.10 ± 0.51 mm***
c	*Poria Cocos* + *Glycyrrhizae Radix *	48.58 ± 1.42 mm
d	*Atractylodis Rhizoma* + *Glycyrrhizae Radix *	20.29 ± 0.69 mm*
e	*Radix Ginseng* + *Glycyrrhizae Radix *	15.32 ± 0.64 mm**
f	*Radix Ginseng* + *Poria Cocos *	21.50 ± 0.55 mm*
g	*Radix Ginseng* + *Atractylodis Rhizoma *	41.59 ± 1.06 mm
h	*Atractylodis Rhizoma* + *Poria Cocos *	34.99 ± 0.96 mm
i	*Glycyrrhizae Radix *+ *Poria coco*s + *Atractylodis Rhizoma *	23.85 ± 0.31 mm
j	*Radix Ginseng* + *Poria Cocos* + *Glycyrrhizae Radix *	46.78 ± 0.53 mm
k	*Radix Ginseng* + *Poria Cocos* + *Atractylodis Rhizom*a	15.94 ± 0.29 mm*
l	*Radix Ginseng* + *Atractylodis Rhizom*a + *Glycyrrhizae Radix *	114.66 ± 1.33 mm

*P < 0.05; **P < 0.01; ***P < 0.001; *N* = 6; mean (mm) ± S.E.

**Table 3:** Therapeutic effects of continuous administration of SJZT on peptic ulcers.

Models	Ulcerative length	Ratio	Amount
Ulcer induced by water immersion for 7 days	120.17 ± 2.23 mm	100%	*N* = 20
SJZT (500 mg/kg) P.O. for 14 days	97.50 ± 1.88 mm	81.25%	*N* = 10
SJZT (500 mg/kg) P.O. for 21 days	78.67 ± 1.47 mm*	65.47%	*N* = 10

*P < 0.05; Mean (mm) ± S.E.; P.O. means per os or oral administration; Control group: Ulcer induced by water immersion for 7 days.

**Table:** (a)

Brain	Reagents	N A	D A	5-HT
Cortex	Dist. H_2_O 5.0 mL/kg	40.9 ± 2.3	102.4 ± 3.7	50.3 ± 2.2
	SJZT 500 mg/kg	31.3 ± 1.4	86.2 ± 3.3	34.1 ± 4.9
	SJZT1000 mg/kg	17.3 ± 1.1*	62.8 ± 5.6*	25.9 ± 2.8*

Brain stem	Dist. H_2_O 5.0 mL/kg	47.9 ± 0.9	131.7 ± 3.4	57.2 ± 3.1
	SJZT 500 mg/kg	41.6 ± 0.3	79.4 ± 1.9*	39.2 ± 0.8*
	SJZT 1000 mg/kg	35.6 ± 0.5	77.8 ± 2.1*	35.1 ± 1.8*

*P < 0.05; mean ± S.E.;  *N* = 6; peroral, ng/g (wet weight); control group: Dist. H_2_O 5.0 mL/kg (B.W.).

**Table:** (b)

Brain	Reagents	HVA	5-HIAA	VMA
Cortex	Dist. H_2_O 5.0 mL/kg	7.2 ± 0.4	48.2 ± 2.5	10.2 ± 0.9
	SJZT 500 mg/kg	8.1 ± 0.2	48.2 ± 1.7	10.9 ± 1.8
	SJZT1000 mg/kg	6.8 ± 0.3	21.3 ± 0.8*	10.1 ± 1.3

Brain stem	Dist. H_2_O 5.0 mL/kg	7.2 ± 0.4	48.2 ± 2.5	10.2 ± 0.9
	SJZT 500 mg/kg	7.8 ± 0.7	80.4 ± 1.6*	8.9 ± 0.2
	SJZT 1000 mg/kg	6.6 ± 0.4	73.8 ± 1.0	7.6 ± 0.3

*P < 0.05; mean ± S.E.; *N* = 6; peroral, ng/g (wet weight).
